# The Influences of Pore Blockage by Natural Organic Matter and Pore Dimension Tuning on Pharmaceutical Adsorption onto GO-Fe_3_O_4_

**DOI:** 10.3390/nano13142063

**Published:** 2023-07-13

**Authors:** Ming-Cyuan He, Sian-Jhang Lin, Tao-Cheng Huang, Guan-Fu Chen, Yen-Ping Peng, Wei-Hsiang Chen

**Affiliations:** 1 Institute of Environmental Engineering, National Sun Yat-sen University, Kaohsiung 804, Taiwan; tryit047607@gmail.com (M.-C.H.); lin820815@gmail.com (S.-J.L.); haso5438@gmail.com (T.-C.H.); kfchan64@gmail.com (G.-F.C.); yppeng@mail.nsysu.edu.tw (Y.-P.P.); 2 Aerosol Science and Research Center, National Sun Yat-sen University, Kaohsiung 804, Taiwan; 3 Department of Public Health, Kaohsiung Medical University, Kaohsiung 807, Taiwan; 4 Center for Emerging Contaminants Research, National Sun Yat-sen University, Kaohsiung 804, Taiwan

**Keywords:** graphene oxide, iron oxide, selective adsorption, pharmaceuticals, pore blockage, natural organic matter

## Abstract

The ubiquitous presence of pharmaceutical pollution in the environment and its adverse impacts on public health and aquatic ecosystems have recently attracted increasing attention. Graphene oxide coated with magnetite (GO-Fe_3_O_4_) is effective at removing pharmaceuticals in water by adsorption. However, the myriad compositions in real water are known to adversely impact the adsorption performance. One objective of this study was to investigate the influence of pore blockage by natural organic matter (NOM) with different sizes on pharmaceutical adsorption onto GO-Fe_3_O_4_. Meanwhile, the feasibility of pore dimension tuning of GO-Fe_3_O_4_ for selective adsorption of pharmaceuticals with different structural characteristics was explored. It was shown in the batch experiments that the adsorbed pharmaceutical concentrations onto GO-Fe_3_O_4_ were significantly affected (dropped by 2–86%) by NOM that had size ranges similar to the pore dimensions of GO-Fe_3_O_4_, as the impact was enhanced when the adsorption occurred at acidic pHs (e.g., pH 3). Specific surface areas, zeta potentials, pore volumes, and pore-size distributions of GO-Fe_3_O_4_ were influenced by the Fe content forming different-sized Fe_3_O_4_ between GO layers. Low Fe contents in GO-Fe_3_O_4_ increased the formation of nano-sized pores (2.0–12.5 nm) that were efficient in the adsorption of pharmaceuticals with low molecular weights (e.g., 129 kDa) or planar structures via size discrimination or inter-planar π-π interaction, respectively. As excess larger-sized pores (e.g., >50 nm) were formed on the surface of GO-Fe_3_O_4_ due to higher Fe contents, pharmaceuticals with larger molecular weights (e.g., 296 kDa) or those removed by electrostatic attraction between the adsorbate and adsorbent dominated on the GO-Fe_3_O_4_ surface. Given these observations, the surface characteristics of GO-Fe_3_O_4_ were alterable to selectively remove different pharmaceuticals in water by adsorption, and the critical factors determining the adsorption performance were discussed. These findings provide useful views on the feasibility of treating pharmaceutical wastewater, recycling valuable pharmaceuticals, or removing those with risks to public health and ecosystems.

## 1. Introduction

Pharmaceuticals are one example of contaminants of emerging concern (CECs)m referring to the chemicals which are not frequently monitored but are under examination for future regulation. These chemicals have attracted increasing concern due to their ubiquitous occurrences in the environment and potential adverse impacts on public health and aquatic ecosystems [1]. Although certain pharmaceuticals are biodegradable and some are excreted from human bodies as degradation intermediates [2], the continuously increasing use of pharmaceuticals has led to their pseudo-persistence in the environment [3]. In recent decades, many studies have investigated pharmaceutical pollution in different water environments. For example, Robert et al. analyzed the concentrations of 11 pharmaceuticals in Australia’s largest inland sewage treatment plant and reported that the removal of most pharmaceuticals in the plant was incomplete [4]. Certain pharmaceuticals are known to form carcinogenic byproducts during disinfection [5,6]. A study that reviewed the current research trends on pharmaceutical pollution suggested that these chemicals are limitedly biodegradable, and integrated/hybrid technologies are recommended for their removal from wastewater [7]. Concentrations of pharmaceuticals at trace levels from ng/L to mg/L in various environmental compartments have been documented [8,9].

Pharmaceuticals include many compounds with different structural characteristics and physicochemical properties. Metformin (MET) is a medication widely used in the treatment of diabetes and has been the most-used pharmaceutical in Taiwan for many years (more than 720 tons in 2019) [5]. Its absorption uptake rate in the human body is approximately 70%, as most of the remaining portion in excreted urine is typically unchanged [10]. MET was observed in more than 90% of samples in a study that monitored the surface water quality in North China [11]. MET was detected in the influents of wastewater treatment plants in the US with high abundance, from several ng/L to less than a hundred mg/L [12]. Diclofenac (DCF) is another pharmaceutical that has a moderate absorption uptake rate (65%) and commonly remains unchanged in excreted urine [13]. It is a widely used non-steroid anti-inflammatory drug (NSAID) and has negative influences on aquatic life with continuous exposure even at trace levels [14]. Although high DCF concentrations up to a range of mg/L were reported in rivers and wastewater [13], it is photochemically active, resulting in potential degradation in the environment [14]. Propranolol (PRO) is a beta-blocker medication typically used in the treatment of hypertension and angina. Over 80% of the intake dose can be excreted from the human body after metabolism [15]. As a result, PRO is frequently detected in rivers [16,17] and estuaries [18] in concentrations of up to 142 ng/L.

Graphene is a two-dimensional (2-D) allotrope of carbon which is an excellent thermal and electrical conductor with fine mechanical strength [19]. Its hydrophobic nature sometimes hinders the application of graphene in water and wastewater treatment. Oxidation of graphene surface forms graphene oxide (GO) enriched with oxygen-containing functional groups that retain the layered structures and are stably dispersed in water [20]. The hydrophilic GO has a large surface-area-to-volume ratio by being spread as layers of hexagonal ring-bound carbon structure, causing all atoms in the sheets to be exposed in water. These characteristics make this material a possible adsorbent for pollutant removal in water. Previously, we synthesized GO coated with magnetite (GO-Fe_3_O_4_) that was capable of removing pharmaceuticals in deionized water [21] and real wastewater [22]. Compared to GO, the GO-Fe_3_O_4_ composite is more readily removed from the water after adsorption by using a magnetic force. The distance between GO layers and the associated surface area was adjustable by controlling the mass ratio of Fe_3_O_4_ to GO in the synthesis of the composite. It has been reported that Fe_3_O_4_ strengthened the van der Waals force and π-π interaction between GO layers, preventing aggregation and thus increasing surface area and adsorption capacity [21,23].

Studies have reported efficient adsorption of organic and inorganic pollutants including pharmaceuticals onto GO-Fe_3_O_4_ for their removals from the water phase [24,25,26]. However, while adsorption represents a cost-effective option with high efficiency to remove trace pollutants in water and wastewater [27], the myriad compositions in real water bodies are expected to adversely impact the performance of this technology [28]. Our previous study has observed that the natural organic matter (NOM) in municipal wastewaters reduced the adsorption of chlorpheniramine (CLP; an antihistamine medication that treats upper respiratory infection and allergic conditions in human health) on GO-Fe_3_O_4_ by approximately 30% [29]. The major constituents in NOM are humic and fulvic acids that have carboxylic and phenolic groups and different molecular sizes [30]. As such, one objective of this study was to extend our previous findings and to investigate whether the size of NOM is important to affect the pharmaceutical adsorption onto GO-Fe_3_O_4_, discussing if the impact stemmed from the competition for adsorption sites in the pores of GO-Fe_3_O_4_. Additionally, as the pore volume and size distribution are adjustable by changing the Fe content in the synthesis of GO-Fe_3_O_4_ [21,24], we further explored the feasibility of altering the surface characteristics of GO-Fe_3_O_4_ to selectively remove different pharmaceuticals in water by adsorption and the critical factors determining the adsorption performance.

## 2. Materials and Methods

### 2.1. Materials

Graphite powder (>99.95%; Acros Organics, Waltham, MA, USA), ferrous chloride (FeCl_2_; Thermo Fischer Scientific, Waltham, MA, USA), and ferric chloride (FeCl_3_; Thermo Fischer Scientific, Waltham, MA, USA) were used for the preparation of GO-Fe_3_O_4_. Standards of pharmaceuticals, including chlorpheniramine maleate, metformin hydrochloride, diclofenac sodium, and propranolol hydrochloride, as well as hydrogen peroxide (H_2_O_2_), hydrogen chlorite (HCl), and ammonium chloride (NH_4_Cl), were purchased from Sigma-Aldrich (St. Louis, MO, USA). The Suwannee-River-derived NOM (SWNOM) standard was obtained from the International Humic Substances Society (IHSS, Monterey Park, CA, USA). The dialysis membrane (Cellu-Sep, Chicago, IL, USA) was used for the preparation of GO. Potassium permanganate (KMnO_4_) and sodium nitrate (NaNO_3_) were obtained from J.T. Baker (Phillipsburg, NJ, USA). Methanol (J.T. Baker, Phillipsburg, NJ, USA) and acetone (Avantor, Radnor, PA, USA) were used as the solvents. The buffers included sodium dihydrogen phosphate (NaH_2_PO_4_; Avantor, Radnor, PA, USA), sodium acetate (CH_3_COONa; Sigma-Aldrich, St. Louis, MO, USA), sodium bicarbonate (NaHCO_3_; Sigma-Aldrich, St. Louis, MO, USA), and sodium carbonate (Na_2_CO_3_; Sigma-Aldrich, St. Louis, MO, USA). Sodium hydroxide (NaOH; Uniregion Biotech, Pomona, CA, USA) and sulfuric acid (H_2_SO_4_; Sigma-Aldrich, St. Louis, MO, USA) were used to adjust the pH in the experiments.

### 2.2. GO-Fe_3_O_4_ Synthesis

The GO-Fe_3_O_4_ synthesis was modified from our previously published studies [24,31]. The surface characteristics of the GO-Fe_3_O_4_ composites prepared in this and other studies were similar, as discussed below, suggesting the reproducibility of the samples. Before the synthesis of GO-Fe_3_O_4_, GO was prepared. Graphite (1 g) was mixed with NaNO_3_ (0.5 g) in H_2_SO_4_ (23 mL) for 15 min. KMnO_4_ (3 g) was slowly added for 60 min. The temperature of the solution was controlled at 35–40 °C for 2 h, followed by the addition of water (46 mL). After the color of the solution was changed from gray to brown, the solution was heated to 85–90 °C for 15 min. When the solution was cooled again, H_2_O_2_ (30%; 10 mL) was added to turn the solution color yellow. The solid in the solution was filtered and mixed with HCl (10% *v*/*v*). The solid in the solution was collected again and mixed with HCl (12.5% *v*/*v*). The final solution was moved to the dialysis membrane (molecular weight cutoff (MWCO): 6000–8000, Scientific Biotech Corp., Taipei, Taiwan) and placed in deionized water. When the solution color in the membrane became black, the solution was ultrasonicated for 30 min and centrifuged (2000 rpm) for 10 min to obtain GO.

Before the synthesis of GO-Fe_3_O_4_, Fe_3_O_4_ was prepared by mixing FeCl_3_ and FeCl_2_ with a ratio of 2.6 on a weight basis (the molar ratio of Fe^3+^ to Fe^2+^ was 2). GO (40 mg; dry weight) was added into different volumes of Fe_3_O_4_ to form GO-Fe_3_O_4_ with different Fe_3_O_4_/GO mass ratios. The solution was heated to 85 °C, and NH_4_Cl (25%) was added to adjust the solution pH to 10. The solution was then rapidly stirred for 45 min to ensure a complete reaction. After the solution was cooled, the solids in the solution were collected by centrifugation and washed with deionized water. The GO-Fe_3_O_4_ composite was then obtained by drying the solids at 70 °C. The names of GO-Fe_3_O_4_, such as GO-Fe_3_O_4_-2.5, GO-Fe_3_O_4_-18, and GO-Fe_3_O_4_-72, in the following discussion denote the composites prepared with FeCl_3_-to-GO ratios of 2.5, 18, and 72 on a weight basis, respectively. All GO-Fe_3_O_4_ composites used in the experiments were controlled at the 40 mesh size.

### 2.3. NOM Fractionation

Two NOM sources, the SWNOM standard provided by the IHSS and the influent of a local wastewater treatment plant in southern Taiwan (LNOM), were used to investigate their interferences on pharmaceutical adsorption onto GO-Fe_3_O_4_ in water. The fractionation of the two NOM sources was modified from the published procedure [32]. The NOM standards were dissolved in deionized water, followed by adjusting the pH to 7. The solution was continuously stirred overnight and filtered to remove undissolved solids. A series of Jumbosep centrifugal filters (Pall Corp., New York, NY, USA) with decreasing MWCO of 60, 18, and 6 kDa were used for NOM fractionation. Before the fractionation, the filter units were pre-rinsed with deionized water and centrifuged multiple times. The NOM solutions were added to the filter units and centrifuged with 4500 rounds per min (rpm) for 60 min. Sequential fractionation was conducted to acquire NOM fractions with >60 kDa, (18–60) kDa, (6–18) kDa, and eventually <6kDa. Simply speaking, the fractionation started by using the filters with an MWCO of 60 kDa. The filtrate from a higher MWCO was used in the following stage of sequential filtration. The total organic carbon (TOC) concentrations and ultraviolet absorbance at a wavelength of 254 nm (UV_254_) of the retentates from different stages of sequential filtration were analyzed by using a TOC analyzer (1030W, Aurora, CO, USA) and a UV-Visible scanning spectrophotometer (DR6000; HACH, Loveland, CO, USA), respectively. The specific ultraviolet absorbance (SUVA), which is the ratio of UV254 to TOC, was calculated to indicate the composition of NOM concerning the hydrophobicity, molecular characteristics, and aromaticity [33]. All NOM solutions after fractionation were stored in the dark at 4 °C.

### 2.4. Characterization

The surface morphology of the composites was analyzed with a scanning electron microscope (Zeiss Supra 55, Jena, Germany). The crystalline structures were determined by using an X-ray diffractometer (Bruker D8, Ettlingen, Germany) with monochromatic Cu-Kα radiation (λ = 1.542 Å). The thermal behaviors of the composites were examined by using Pyris 1 thermogravimetric analyzer (PerkinElmer, Waltham, MA, USA) at a heating rate of 10 °C/min in a temperature range from 30 °C to 875 °C. The surface zeta potentials at different pHs were investigated using the Zeta Potential measurement system (Malvern Zetasizer Nano Range, Malvern, UK). The specific surface areas were calculated by using the Brunauer–Emmett–Teller (BET) theory, and the pore size distributions and pore volumes were calculated with the N_2_ adsorption–desorption isotherms based on Barrett–Joyner–Halenda (BJH) theory using an adsorption analyzer (Micromeritics ASAP 2020, Norcross, GA, USA).

### 2.5. Adsorption Experiment

**Figure 1:**
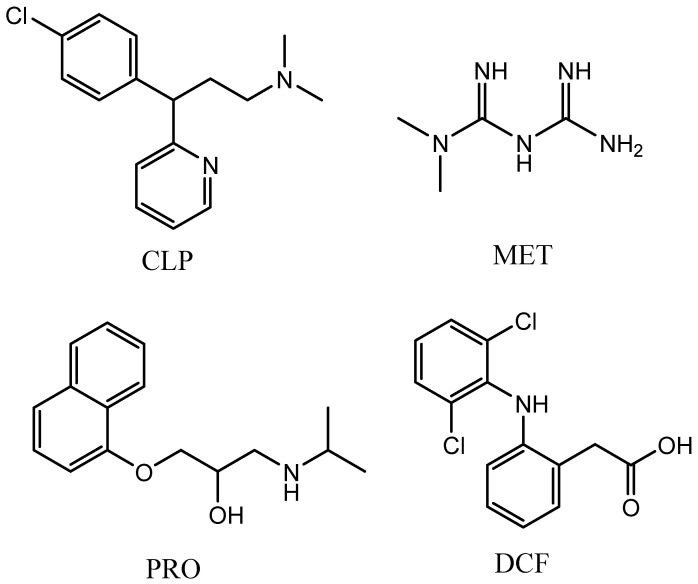
Chemical structures of CLP, MET, PRO, and DCF.

Batch experiments were conducted in 50 mL polyethylene centrifuge tubes. In the first experiments, CLP (Figure 1) was used as the model adsorbate. The effects of two NOM solutions with different sizes and different reaction pHs on CLP adsorption onto GO-Fe_3_O_4_ were investigated. In the next experiments, three different pharmaceuticals, MET, DCF, and PRO, were selected as the target adsorbates (Figure 1) given their different molecular sizes and structural characteristics. In all experiments, after the reactions were at equilibrium, GO-Fe_3_O_4_ was removed from the water phase via centrifugation (4000 rpm for 15 min) and magnetic separation. The pharmaceutical concentrations in water were sampled and analyzed at least in duplicate and within 6 h to limit the influence of water quality variation after the experiments. The pharmaceutical concentrations in water after the experiments were used to calculate the adsorbed concentrations on the surface of GO-Fe_3_O_4_ (q_e_).

### 2.6. Pharmaceutical Analysis

The pharmaceutical analysis was modified from our previously published studies [5,24]. The concentrations of pharmaceuticals in the experiments were pre-treated by solid-phase extraction (SPE), followed by the analysis using ultra-high-pressure liquid chromatography coupled with a tandem mass spectrometer (UPLC-MSMS) [29]. The extraction cartridge (Oasis HLB, Waters Corp, Milford, MA, USA) used in SPE consists of a polypropylene tube (3 mL) packed with hydrophilic polymer (60 mg). Before the extraction, the column was pre-treated with ethyl acetate (3 mL), methanol (3 mL), and deionized water (3 mL) in order with a flow rate of 3 mL/min. After the column was dried, 250 mL of water sample was introduced into the SPE column by vacuum. Next, 3 mL of ethyl acetate was used to elute pharmaceuticals adsorbed in the column. 1-Hydroxypyren (100 ng/L, 10 μL) (Sigma-Aldrich, St. Louis, MO, USA) was spiked as a surrogate into the ethyl acetate extract. The UPLC (Agilent 1290 II, Santa Clara, CA, USA) was equipped with a 4.6 mm × 150 mm Poroshell 120 EC-C18 column (2.7 µm particle size; Agilent, Santa Clara, CA, USA), and 1 mL of the extract was injected. The mobile phase consisted of acetonitrile (60%, *v*/*v*) and ultrapure water (40%, *v*/*v*/) with 0.1% formic acid added. The flow rate was 0.5 mL/min. The column temperature was 40 °C. The MSMS (Agilent 5430, Santa Clara, CA, USA) was performed in the multiple reaction monitoring mode (MRM) with an ion source of electrospray ionization (ESI).

### 2.7. Adsorption Isotherm and Kinetics

Adsorption isotherm and kinetics are commonly used to fit the experimental adsorption data of compounds in the water phase. Two isotherm models, the Langmuir (Equation (1)) and Fruendlich isotherms (Equation (2)), were applied to determine the isotherm parameters of different pharmaceutical adsorption onto GO-Fe_3_O_4_ as follows.

(1) $$\frac{C}{q_{e}}=\frac{1}{K_{L}q_{max}}+\frac{C}{q_{m}}$$

(2) $$logq_{e}=logK_{F}+n\times logC$$

where C [mg/L] and q_m_ [mg/g] represent the adsorbate concentrations in the water phase and adsorbed on the surface of the adsorbent, respectively; q_max_ [mg/g] denotes the maximum adsorbed concentration in the Langmuir model; K_L_ [L/mg] and K_F_ [(mg/g)/(mg/L)^n^] represent the Langmuir constant and Freundlich capacity factor, respectively; and n denotes the Freundlich exponent. The kinetics of different pharmaceutical adsorption onto GO-Fe_3_O_4_ was studied as well. By fitting different kinetic models, the pseudo-second-order model, which is expressed as follows, yielded the best fit for the observation.

$$\frac{t}{q_{t}}=\frac{1}{k{q_{e}}^{2}}+\frac{t}{q_{e}}$$

where k represents the second-order rate constant [g/mg-min]; and q_t_ and q_e_ denote the adsorbed concentrations onto GO-Fe_3_O_4_ at the time t and equilibrium [mg/g], respectively.

## 3. Results and Discussion

### 3.1. Surface Characterization of GO-Fe_3_O_4_

**Figure 2:**
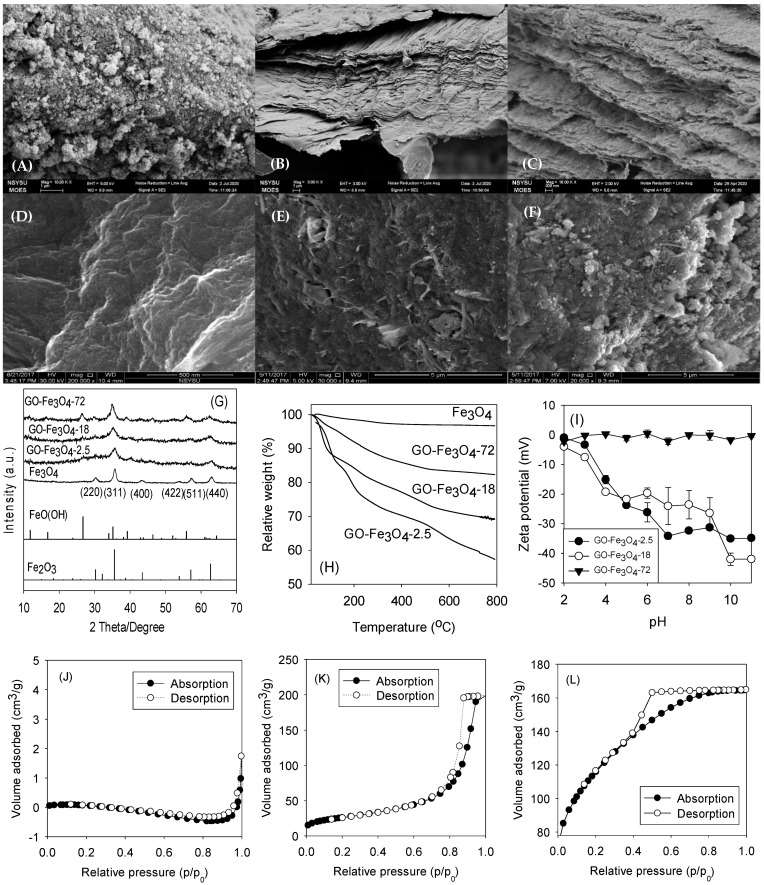
(**A**) SEM images of the side views of GO, (**B**) Fe_3_O_4_, and (**C**) GO-Fe_3_O_4_-2.5; (**D**) front views of GO-Fe_3_O_4_-2.5, (**E**) GO-Fe_3_O_4_-18, and (**F**) GO-Fe_3_O_4_-72; (**G**) XRD patterns; (**H**) TGA results; (**I**) surface charge variations of GO-Fe_3_O_4_ at different pHs; and (**J**) low-temperature N_2_ adsorption/desorption isotherms of GO, (**K**) Fe_3_O_4_, and (**L**) GO-Fe_3_O_4_-2.5.

The morphologies of Fe_3_O_4_, GO, and GO-Fe_3_O_4_ in side view were investigated by scanning electron microscopy (SEM), as shown in Figure 2A–C. While Figure 2A reveals the complex Fe_3_O_4_ crystalline aggregates, single or multiple layers of tightly stacked GO are shown in Figure 2B. As shown in Figure 2C, as micro-sized aggregates of Fe_3_O_4_ were dispersed and anchored between the GO layers, the distances between the GO curtains were increased. The morphology of GO-Fe_3_O_4_ still showed a laminated state but became hybrid. Figure 2D–F show the images of GO-Fe_3_O_4_ prepared with different Fe_3_O_4_/GO ratios in the front view. When the Fe fraction in the composite was increased, larger-sized Fe_3_O_4_ aggregates caused the phenomenon of the layer-stacking GO-Fe_3_O_4_ structure to become more obscure [21,24]. Figure 2G shows the X-ray diffraction (XRD) results. The crystalline characteristics of GO-Fe_3_O_4_-2.5, GO-Fe_3_O_4_-18, and GO-Fe_3_O_4_-72 at 2θ peaks of 30.34°, 35.73°, 43.24°, 54.01°, 57.40°, and 62.97° were assigned to the lattice planes of (220), (311), (400), (422), (511), and (440) (JCPDS No. 89-3854), respectively [25]. These peaks were intensified by increasing the Fe fraction in the composite. A peak at 2θ 10.30° was found in the result of GO-Fe_3_O_4_ due to the interplanar spacing of GO [21]. A peak at 2θ 27.73° representing the occurrence of FeO(OH) was observed in the profile of GO-Fe_3_O_4_ and increased when the Fe_3_O_4_ fraction was increased.

Figure 2H shows the thermogravimetric analysis (TGA) results generated on GO-Fe_3_O_4_-2.5, GO-Fe_3_O_4_-18, and GOFe_3_O_4_-72. The percent loss in mass of GO-Fe_3_O_4_ as a function of temperature was illustrated. The curves showed that maximum oxidation of carbon took place between 600 °C and 650 °C. Studies have reported similar temperature ranges for carbon oxidation [34,35]. The residual weights were associated with the metal contents of the composites. More mass losses were found as the GO content of the GO-Fe_3_O_4_ composites was increased. The total mass losses of 40%, 28%, 23%, and 15% were found and corresponded with the GO contents of the composites prepared with different Fe_3_O_4_/GO ratios, respectively. The zeta potential variations of GO-Fe_3_O_4_ prepared with different Fe fractions were analyzed and are shown in Figure 2H. The zeta potential of GO-Fe_3_O_4_-2.5 ranged from −35 to 0 mV. It was reported that the colloidal dispersion became stable if the zeta potential was above +30 mV or below −30 mV [36], such as in the cases of GO-Fe_3_O_4_-2.5 and GO-Fe_3_O_4_-18 at high pH values. The pH of zero charges (pH_zvc_) that showed the maximum coagulation and flocculation occurred at pH 2 in the cases of GO-Fe_3_O_4_-2.5 and GO-Fe_3_O_4_-18, suggesting these composites resisted aggregation at neutral and higher pHs. A high Fe fraction (GO-Fe_3_O_4_-72) neutralized the negative zeta potential and reduced the electrostatic repulsion between GO layers (~0 mV from pH 2 to 11). These observations suggest that GO-Fe_3_O_4_-72 could be aggregated at different pHs for easier separation from the water after adsorption, whereas GO-Fe_3_O_4_-2.5 and GO-Fe_3_O_4_-18 were well dispersed for potentially better adsorption at neutral and high pHs.

**Figure 3:**
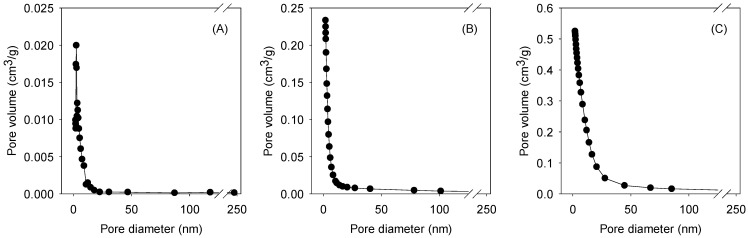
Pore size distributions of (**A**) GO-Fe_3_O_4_-2.5, (**B**) GO-Fe_3_O_4_-18, and (**C**) GO-Fe_3_O_4_-72.

Table 1 lists the specific surface areas and pore volume distributions of GO-Fe_3_O_4_ prepared with different Fe_3_O_4_/GO ratios analyzed by the Brunauer–Emmett–Teller (BET) theory. The results revealed that the pore sizes of the GO-Fe_3_O_4_ composites were mostly below 50 nm and the total pore volume decreased from 1.09 (GO-Fe_3_O_4_-72) to 0.21 cm^3^/g (GO-Fe_3_O_4_-2.5). Nevertheless, as the Fe fraction was reduced, the total specific surface area was increased by 34.7% from 302 (GO-Fe_3_O_4_-72) to 407 m^2^/g (GO-Fe_3_O_4_-2.5). The internal surface area was more significantly increased by 585.7% from 14 (GO-Fe_3_O_4_-72) to 96 m^2^/g (GO-Fe_3_O_4_-2.5). Zhao et al. reported the surface area (84.091 m^2^/g) and pore volume (0.246 cm^3^/g) of Fe_3_O_4_ nanoparticles [37]. Alizadeh et al. observed a pore volume of 0.0763 cm^3^/g for Fe_3_O_4_ through the N_2_ adsorption–desorption measurement [38]. The higher surface area and the dominance of micro-/mesopores could improve the capture and removal of compounds in water by size discrimination or capillary force. Figure 3 shows the particle size distribution analyses of the composites, indicating that most of the pore sizes were below 50 nm, and ~90% of the pore sizes of GO-Fe_3_O_4_-2.5 ranged from 1 to 7 nm.

### 3.2. Pore Blockage Effect of NOM

**Figure 4:**
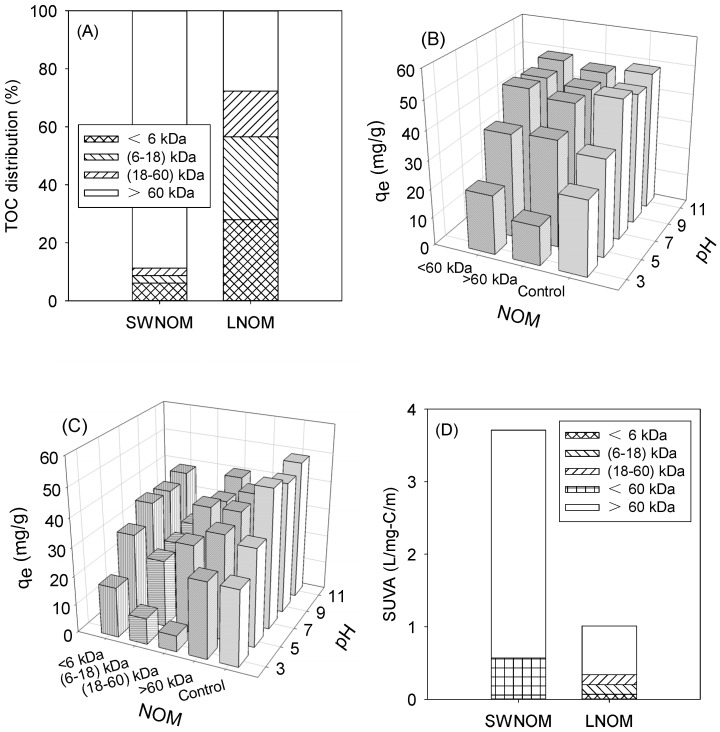
(**A**) TOC analyses of SWNOM and LNOM, pore blockage effects by (**B**) SWNOM and (**C**) LNOM on CLP adsorption onto GO-Fe_3_O_4_ at different pHs, and (**D**) SUVA analyses of SWNOM and LNOM. The CLP and GO-Fe_3_O_4_ concentrations were 20 and 400 mg/L, respectively. The reaction time was 24 h.

The successful sequential fractionation of SRNOM and LNOM to produce distinct molecular weight (MW) fractions was confirmed by TOC analysis, as shown in Figure 4A. The NOM recoveries based on the cumulative TOC concentrations through the entire sequential fractionation ranged from 77% to 95%. In Figure 4A, the fractions of SRNOM <6 kDa, (6–18) kDa, (18–60) kDa, and >60 kDa are 6%, 3%, 3%, and 89%, respectively. The fractions of LNOM < 6 kDa, (6–18) kDa, (18–60) kDa, and >60 kDa were 28%, 29%, 16%, and 28%, respectively. The larger-sized OM (>60 kDa) dominated the SRNOM, whereas the sizes of OM in the LNOM were more equally distributed. Given this observation, the SRNOM fractions >60 and <60 kDa were separated and used to investigate their impacts on the adsorption performance of GO-Fe_3_O_4_.

Figure 4B reveals the pore blockage of SRNOM with different sizes (20 mg/L) on CLP adsorption onto GO-Fe_3_O_4_. The adsorbed concentration was estimated by calculating the CLP concentration adsorbed onto GO-Fe_3_O_4_. In the results, the pH effect seemed to be more critical than the NOM size for influencing the adsorption performance. Given the pKa values of CLP (3.7 and 9.2) [21] and zeta potential of GO-Fe_3_O_4_ (Figure 2I), neutral or high pHs resulted in stronger electrostatic attraction between positive CLP and negative GO-Fe_3_O_4_, increasing the adsorption efficiency. Compared with the result of the control experiment, the adsorbed concentration dropped by up to 48% in the presence of NOM. Humic acid and fulvic acid, two common species of NOM, typically exhibit negative surface charges at neutral and high pHs [39,40], and are expected to have a lower degree of interference on CLP adsorption onto GO-Fe_3_O_4_ due to the electrostatic repulsion. However, the repulsion force decreased at low pHs, increasing the pore blockage effect of NOM. At a low pH (e.g., pH 3 in Figure 4B), evident competitive adsorption between CLP and NOM, notably those with MW > 60 kDa, onto GO-Fe_3_O_4_ was observed.

Figure 4C shows the pore blockage of LNOM with different sizes (20 mg/L) on CLP adsorption onto GO-Fe_3_O_4_. Like the observation using SRNOM, the adsorbed concentration was significantly reduced at low pHs in the absence and presence of NOM with different sizes (the q_e_ was dropped by 2–86% from pH 11 to 3). However, besides the pH effect, it was found that the NOM between (6–18) kDa exhibited higher impacts on the adsorption at neutral and high pHs, as the influence of NOM between (6–18) kDa and (18–60) kDa became more evident at pH 3 (the adsorbed concentration was dropped by 66% and 86%, respectively). Using an equation of R_min_ = (3V/4)^1/3^ (where R_min_ and V represent the size in nm and MW in Da, respectively) for the NOM size prediction [41], the size ranges of NOM between (6–18) kDa and (18–60) kDa were (1.2–1.7) and (1.7–2.6) nm, respectively. Our BET analysis indicated that ~90% of the pore sizes of GO-Fe_3_O_4_-2.5 ranged from 1 to 7 nm (Figure 3A). Given the similar findings between the pore size estimation and BET analysis, the adsorbed concentration was significantly affected by the presence of NOM that had size ranges close to the pore dimensions of GO-Fe_3_O_4_, especially when adsorption occurred at low pHs. Similar pore blockage effects of NOM or minerals on the adsorption of different compounds have also been reported [42,43]. Figure 4D shows the SUVA analyses of SWNOM and LNOM. The SUVA values of SWNOM < 60 and > 60 kDa were 0.57 and 3.14 L mg-C^−1^ m^−1^, respectively. The SUVA values of LNOM fractions < 6, (6–18), (18–60), and >60 kDa were 0.07, 0.13, 0.14, and 0.67 mg-C^−1^ m^−1^, respectively. Although the SWNOM, notably the fraction > 60 kDa, exhibited higher hydrophobicity and aromaticity, given the observations above, the pore size effect of NOM appeared to be more critical in interfering with the CLP adsorption onto GO-Fe_3_O_4_.

### 3.3. Selective Adsorption by Pore Size Tuning: Isotherm

**Figure 5:**
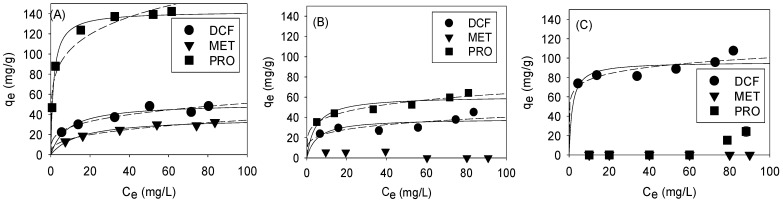
Pharmaceuticals adsorptions onto (**A**) GO-Fe_3_O_4_-2.5, (**B**) GO-Fe_3_O_4_-18, and (**C**) GO-Fe_3_O_4_-72. The solid and dashed lines denote the Langmuir and Freundlich fits of the observed data, respectively. The initial pharmaceutical concentration ranged from 10 to 90 mg/L. The experimental pH was 6 and the contact time was 24 h. The fitting was not conducted for certain results that showed negligible adsorbed concentrations.

While pore blocking that occurred when NOM exhibited size distributions similar to those of GO-Fe_3_O_4_-influenced CLP adsorption, the feasibility of tuning the pore size dimension of GO-Fe_3_O_4_ for selective pharmaceutical adsorption was investigated. The specific surface area, pore volume, and pore size distribution of GO-Fe_3_O_4_ were adjusted by changing the Fe_3_O_4_/GO ratio in the synthesis procedure (Table 1). Figure 5A–C reveal the adsorption of MET, PRO, and DCF onto these GO-Fe_3_O_4_ composites. The adsorbed concentrations of pharmaceuticals quickly increased and reached apparent plateaus. In the result of the GO-Fe_3_O_4_-S2.5 experiment, the maximum adsorption capacities among three pharmaceuticals were decreased in the order: PRO > DCF > MET. A similar order was found in the experiment using GO-Fe_3_O_4_-18. However, when GO-Fe_3_O_4_-72 was added to the experiment, the DCF adsorption dominated and the adsorbed concentrations of the other two pharmaceuticals were negligible.

Our previous study has estimated the crystalline sizes of Fe_3_O_4_ (e.g., 5.60–6.22 nm in GO-Fe_3_O_4_-2.5) using the Scherrer equation and the XRD data, suggesting that the crystalline sizes corresponded to the pore size distributions of GO-Fe_3_O_4_ [21]. As GO-Fe_3_O_4_-2.5 exhibited a relatively higher specific surface area and smaller pore size (Table 1 and Figure 4A), a higher Fe_3_O_4_/GO ratio potentially increased the interplanar spacing and larger-sized pore formation (e.g., GO-Fe_3_O_4_-18 or GO-Fe_3_O_4_-72 in Figure 5B,C, respectively). The MWs of MET, PRO, and DCF were 129, 259, and 296 Da, respectively. Although PRO did not have the lowest MW between the three pharmaceuticals, the fused-ring aromatic (C_10_H_7_O) structure possibly engendered its better adsorption onto GO-Fe_3_O_4_ that was mainly comprised of 2-D GO via size discrimination and/or p-p coupling with benzene rings on the surface of GO layers. In the experiments using GO-Fe_3_O_4_-18 that had a lower specific surface area, the adsorbed concentration of all pharmaceuticals dropped. When the pore size was significantly increased in the case of GO-Fe_3_O_4_-72, DCF that had a larger MW was more easily adsorbed onto GO-Fe_3_O_4_ and dominated the adsorption (Figure 5C).

The Langmuir and Freundlich isotherms were employed to fit the observed data for investigating the adsorption of three pharmaceuticals onto GO-Fe_3_O_4_ (Table 2). The results of MET in the GO-Fe_3_O_4_-18 and GO-Fe_3_O_4_-72 experiments as well as that of PRO in the GO-Fe_3_O_4_-72 experiment were not analyzed, since their adsorbed concentrations were negligible. In the results, the Langmuir model exhibited better fits for all data, indicating monolayer coverage of adsorbed pharmaceuticals on the surface of GO-Fe_3_O_4_ layers [44]. Note that the majority of the surface area was on the GO surface. Adjusting the Fe_3_O_4_/GO ratio changed the interplanar spacing, affecting the ease of reaching the surface area. The decreasing CLP adsorption observed above was attributable to the pore blockage when GO-Fe_3_O_4_ had a pore (or interplanar) size similar to that of NOM.

In Figure 5, PRO and MET showed the highest (144.9 mg/g) and lowest q_max_ (36.8 mg/g) in the GO-Fe_3_O_4_-2.5 experiment. Because of the pKa values of MET (2.8 and 11.5) [45,46] and PRO (9.5) [47,48], their surfaces had positive charges at pH 6 in the experiments [49]. DCF had a negative surface charge at pH 6 due to its pKa value of 4.2 [47,50,51]. Given the negative surface charges of GO-Fe_3_O_4_ (Figure 2I), DCF seemed to be less effectively adsorbed. However, in the experiment using GO-Fe_3_O_4_-72 that had a neutral surface charge because of high Fe content, DCF became the dominant species. The discussion here suggested that, besides the surface area, the structural dimensions (e.g., pore sizes of GO-Fe_3_O_4_ and planarity of PRO) and surface charges of adsorbent and adsorbate were critical and could be tuned for selective adsorption onto GO-Fe_3_O_4_.

### 3.4. Selective Adsorption by Pore Size Tuning: Kinetics

**Figure 6:**
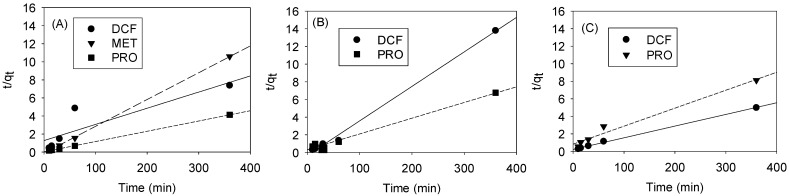
Pseudo-2nd-order linear equations obtained by using the sorption data of pharmaceuticals onto (**A**) GO-Fe_3_O_4_-2.5, (**B**) GO-Fe_3_O_4_-18, and (**C**) GO-Fe_3_O_4_-72.

Figure 6A–C show the experimental data with linear equations of the pseudo-second-order kinetic models obtained by using the linear method for the sorption of three pharmaceuticals under study onto GO-Fe_3_O_4_ prepared with different Fe_3_O_4_/GO ratios. The initial pharmaceutical concentrations were 90 mg/L and the reaction pH was 6. Table 3 lists the values of the pseudo-second-order kinetic model constants (k) and the amount of the pharmaceuticals adsorbed at equilibrium (q_e_). The regression values suggested that there was strong positive evidence that the pharmaceutical adsorption onto GO-Fe_3_O_4_ followed the pseudo-second-order kinetic expression. A pseudo-second-order kinetic model suggested the chemical adsorption of these pharmaceuticals onto GO-Fe_3_O_4_ [52,53]. The initial sorption rates (k × q_e_^2^) [g/mg-min] of three pharmaceuticals onto GO-Fe_3_O_4_ prepared with different Fe_3_O_4_/GO ratios, when t approached zero, were calculated (Table 3). Similar to the trends in Figure 5, the initial adsorption rates of MET (from 8.56 mg/g-min in Figure 6A to negligible in Figure 6B,C) and PRO (from 30.09 in Figure 6A and 3.19 mg/g-min in Figure 6B to 1.21 mg/g-min in Figure 6C) were reduced, as the specific surface area and pore size of GO-Fe_3_O_4_ were decreased and increased, respectively. Note that PRO had a much higher initial adsorption rate (30.09 mg/g min) in the experiment using GO-Fe_3_O_4_-2.5, potentially attributable to its structural planarity enhancing the adsorption onto 2-D GO layers. The increasing initial adsorption rate of DCF (from 2.66 mg/g-min in Figure 6A to 4.47 mg/g-min in Figure 6C) also corresponded well to its more efficient adsorption when GO-Fe_3_O_4_-72 that had larger pore sizes was used (Figure 5C and Figure 6C).

### 3.5. Discussion

Correlation analysis was undertaken to investigate the relationships between the adsorbed concentrations of different pharmaceuticals and surface characteristics of GO-Fe_3_O_4_, as listed in Table 4. A strong negative correlation (r = −0.85) was observed between the Fe content and the specific surface area of GO-Fe_3_O_4_, whereas a higher Fe content increased the surface zeta potential (r = 0.99) and pore volume (r = 0.99). More importantly, the results showed that a lower Fe content in GO-Fe_3_O_4_ increased the adsorption of MET (r = −0.67) and PRO (r = −0.94).

In summary, trace Fe content creating nano-sized Fe_3_O_4_ crystals that prevented GO layer stacking helped form nanopores between Fe_3_O_4_ crystals and GO layers and short interplanar spacing. Smaller compounds, such as MET, or those with 2-D planar structures, such as PRO, that contain nonpolar fused rings at one end of the molecule attached to a chiral side chain could be more efficiently adsorbed onto GO layers under this circumstance by inter-planar π-π interactions or van der Waals forces [54,55]. Increasing the Fe contents enhanced the larger-size pore formation (r = 0.99 in Table 4), attributable to larger sizes of Fe_3_O_4_ crystals and interplanar spacing of GO layers, and increased the surface zeta potential (r = 0.99). Under these circumstances, DCF that had a larger MW (r = 0.98) and a negative surface charge at neutral pHs (r = 0.94) became more effectively adsorbed as observed in the experiments using GO-Fe_3_O_4_-72. The amounts of pharmaceuticals were accommodated in a single molecular layer on the surface of GO-Fe_3_O_4_. The initial chemisorption rates of the pharmaceuticals with structural planarity and those with larger molecular weights were enhanced on the surfaces of GO-Fe_3_O_4_ that were prepared with low and high Fe contents, respectively.

## 4. Conclusions

With the effect of the Fe content on the pore size dimensions of GO-Fe_3_O_4_ and the negative influence of NOM on pharmaceutical adsorption on GO-Fe_3_O_4_, this study determined that the adsorbed pharmaceutical concentrations on the surface of GO-Fe_3_O_4_ were significantly affected by NOM that had size ranges close to the pore dimensions of GO-Fe_3_O_4_. The influence of NOM was further enhanced when the adsorption reaction occurred at low pHs. The pore size dimensions of GO-Fe_3_O_4_ were tunable by changing the Fe content in the synthesis to form the GO-Fe_3_O_4_ composites that had different specific surface areas, zeta potentials, pore volumes, and pore size distributions. As low Fe contents in the synthesis increased the nano-sized pore formation in the GO-Fe_3_O_4_ composite, the adsorption of pharmaceuticals that had smaller sizes (e.g., MET) or planar structural characteristics (e.g., PRO) was enhanced via size discrimination and inter-planar π-π interactions. Increasing the Fe content resulted in excess larger-sized pore formation in the GO-Fe_3_O_4_ composite. In this case, the electrostatic attraction based on the surface charges of adsorbate and GO-Fe_3_O_4_ was critical and the pharmaceuticals with larger MWs became dominant on the surface of the composite.

In conclusion, the presence of pharmaceuticals in wastewater is known as a challenge environmentally, notably due to their unknown impacts on human health and aquatic ecosystems. The findings in this study provided insight into pore blockage by NOM and the potential of tuning the pore dimensions on the surface of GO-Fe_3_O_4_ for selective pharmaceutical adsorption. Although more information on producing and optimizing composites such as GO-Fe_3_O_4_ for real wastewater treatment is needed, the discussion here provided useful views on the feasibility of this technology for treating pharmaceutical wastewater.

## Figures and Tables

**Table 1:** Specific surface areas, pore volumes, and pore size distributions of GO-Fe_3_O_4_ prepared with different Fe_3_O_4_/GO ratios.

Composite	Specific Surface Area (m^2^/g)			Pore Volume			
	Total	Micro	External	Total (cm^3^/g)	<2 nm (%) ^1^	2–50 nm (%) ^1^	>50 nm (%) ^1^
GO-Fe_3_O_4_-2.5	407	96	311	0.21	25	74	1
GO-Fe_3_O_4_-18	331	31	300	0.30	22	77	1
GO-Fe_3_O_4_-72	302	14	288	1.09	7	92	1

^1^ The results listed for <2, 2–50, and >50 nm denote the volumes of micro-, meso-, and macropores in proportion to the total pore volumes of GO-Fe3O4.

**Table 2:** Adsorption isotherm models used to fit the observed sorption data.

		Langmuir			Freundlich		
		q_max_ (mg/g)	K_L_ (L/mg)	R^2^	n	K_F_ ((mg/g)/(mg/L)^n^)	R^2^
GO-Fe_3_O_4_-2.5	PRO	144.93	0.54	0.99	0.23	59.99	0.92
	DCF	51.28	0.11	0.98	0.28	13.95	0.94
	MET	36.76	0.07	0.99	0.36	6.55	0.98
GO-Fe_3_O_4_-18	PRO	66.67	0.12	0.98	0.19	25.69	0.95
	DCF	45.05	0.07	0.89	0.19	16.14	0.65
GO-Fe_3_O_4_-72	DCF	106.38	0.18	0.98	0.10	61.62	0.77

**Table 3:** Adsorption kinetic models used to fit the observed sorption data.

	GO-Fe_3_O_4_-2.5			GO-Fe_3_O_4_-18		GO-Fe_3_O_4_-72	
	MET	PRO	DCF	PRO	DCF	PRO	DCF
k (g/mg min)	7.55 × 10^−3^	3.91 × 10^−3^	1.02×10^−3^	1.01 × 10^−3^	4.56 × 10^−3^	5.1 × 10^−4^	7.9 × 10^−3^
q_e_ (mg/g)	33.67	87.72	51.02	56.18	25.58	48.78	75.19
k × q_e_^2^ (g/mg-min)	8.56	30.09	2.66	3.19	2.98	1.21	4.47
R^2^	0.99	0.99	0.78	0.98	0.99	0.97	0.99

**Table 4:** Correlation coefficients between the surface characteristics of GO-Fe_3_O_4_ and pharmaceutical adsorption. The numbers marked in bold indicate a strong linear relationship (the coefficient is larger than 0.75 or smaller than −0.75).

	GO-Fe_3_O_4_				Adsorbed Concentration		
	Fe Content	SA	ZP	PV	MET	PRO	DCF
Fe content	1				−0.67	**−0.94**	**0.95**
SA	**−0.85**	1			**0.96**	**0.98**	−0.65
ZP	**0.99**	**−0.87**	1		−0.70	**−0.95**	**0.94**
PV	**0.99**	**−0.80**	**0.99**	1	−0.61	**−0.90**	**0.98**

## Data Availability

All the data are available upon request to W.-H. Chen.

## References

[1] Morin-Crini N., Lichtfouse E., Fourmentin M., Ribeiro A.R.L., Noutsopoulos C., Mapelli F., Fenyvesi É., Vieira M.G.A., Picos-Corrales L.A., Moreno-Piraján J.C. (2022). Removal of emerging contaminants from wastewater using advanced treatments. A review. Environ. Chem. Lett..

[2] Su C.C., Cada C.A., Dalida M.L.P., Lu M.C. (2013). Effect of UV light on acetaminophen degradation in the electro-Fenton process. Sep. Purif. Technol..

[3] Nannou C., Ofrydopoulou A., Evgenidou E., Heath D., Heath E., Lambropoulou D. (2020). Antiviral drugs in aquatic environment and wastewater treatment plants: A review on occurrence, fate, removal and ecotoxicity. Sci. Total Environ..

[4] Roberts J., Kumar A., Du J., Hepplewhite C., Ellis D.J., Christy A.G., Beavis S.G. (2016). Pharmaceuticals and personal care products (PPCPs) in Australia’s largest inland sewage treatment plant, and its contribution to a major Australian river during high and low flow. Sci. Total Environ..

[5] Ho M.-C., Yang R.-Y., Chen G.-F., Chen W.-H. (2023). The effect of metformin and drinking water quality variation on haloacetamide formation during chlor(am)ination of acetaminophen. J. Environ. Manag..

[6] Chen W.H., Wang Y.H., Hsu T.H. (2021). The competitive effect of different chlorination disinfection methods and additional inorganic nitrogen on nitrosamine formation from aromatic and heterocyclic amine-containing pharmaceuticals. Chemosphere.

[7] Kumar M., Sridharan S., Sawarkar A.D., Shakeel A., Anerao P., Mannina G., Sharma P., Pandey A. (2023). Current research trends on emerging contaminants pharmaceutical and personal care products (PPCPs): A comprehensive review. Sci. Total Environ..

[8] Surana D., Gupta J., Sharma S., Kumar S., Ghosh P. (2022). A review on advances in removal of endocrine disrupting compounds from aquatic matrices: Future perspectives on utilization of agri-waste based adsorbents. Sci. Total Environ..

[9] O’Connor J., Bolan N.S., Kumar M., Nitai A.S., Ahmed M.B., Bolan S.S., Vithanage M., Rinklebe J., Mukhopadhyay R., Srivastava P. (2022). Distribution, transformation and remediation of poly- and per-fluoroalkyl substances (PFAS) in wastewater sources. Process Saf. Environ. Prot..

[10] Ussery E., Bridges K.N., Pandelides Z., Kirkwood A.E., Bonetta D., Venables B.J., Guchardi J., Holdway D. (2018). Effects of environmentally relevant metformin exposure on Japanese medaka (Oryzias latipes). Aquat. Toxicol..

[11] Kong L., Kadokami K., Wang S., Duong H.T., Chau H.T.C. (2015). Monitoring of 1300 organic micro-pollutants in surface waters from Tianjin, North China. Chemosphere.

[12] Xing Y., Yu Y., Men Y. (2018). Emerging investigators series: Occurrence and fate of emerging organic contaminants in wastewater treatment plants with an enhanced nitrification step. Environ. Sci. Water Res. Technol..

[13] Alessandretti I., Rigueto C.V.T., Nazari M.T., Rosseto M., Dettmer A. (2021). Removal of diclofenac from wastewater: A comprehensive review of detection, characteristics and tertiary treatment techniques. J. Environ. Chem. Eng..

[14] Hlengwa N., Mahlambi P. (2020). SPE-LC-PDA method development and application for the analysis of selected pharmaceuticals in river and wastewater samples from South Africa. Water SA.

[15] Di Lorenzo T., Di Cicco M., Di Censo D., Galante A., Boscaro F., Messana G., Galassi D.M.P. (2019). Environmental risk assessment of propranolol in the groundwater bodies of Europe. Environ. Pollut..

[16] Jiang X.N., Shen Y.T., Wang H.X., Wang C.H., Ye X.X., Xiang Z. (2019). Determination of kaurenoic acid in rat plasma using UPLC-MS/MS and its application to a pharmacokinetic study. J. Pharm. Biomed. Anal..

[17] Guo Y., Guo Z., Zhang L., Yoshimura C., Ye Z., Yu P., Qian Y., Hatano Y., Wang J., Niu J. (2022). Photodegradation of propranolol in surface waters: An important role of carbonate radical and enhancing toxicity phenomenon. Chemosphere.

[18] Zhu F., Yao Z.J., Ji W.L., Liu D.Y., Zhang H., Li A.M., Huo Z.L., Zhou Q. (2020). An efficient resin for solid-phase extraction and determination by UPLCMS/MS of 44 pharmaceutical personal care products in environmental waters. Front. Environ. Sci. Eng..

[19] Sood A.K., Lund I., Puri Y.R., Efstathiadis H., Haldar P., Dhar N.K., Lewis J., Dubey M., Zakar E., Wijewarnasuriya P. (2015). Review of Graphene Technology and Its Applications for Electronic Devices.

[20] Gao W. (2015). The chemistry of graphene oxide. Graphene Oxide.

[21] Li C.M., Chen C.H., Chen W.H. (2017). Different influences of nanopore dimension and pH between chlorpheniramine adsorptions on graphene oxide-iron oxide suspension and particle. Chem. Eng. J..

[22] Lin C.H., Li C.M., Chen C.H., Chen W.H. (2019). Removal of chlorpheniramine and variations of nitrosamine formation potentials in municipal wastewaters by adsorption onto the GO-Fe_3_O_4_. Environ. Sci. Pollut. Res..

[23] Tishbi P., Mosayebi M., Salehi Z., Fatemi S., Faegh E. (2022). Synthesizing magnetic graphene oxide nanomaterial (GO-Fe_3_O_4_) and kinetic modelling of methylene blue adsorption from water. Can. J. Chem. Eng..

[24] Chen W.-H., Huang J.-R., Lin C.-H., Huang C.-P. (2020). Catalytic degradation of chlorpheniramine over GO-Fe_3_O_4_ in the presence of H_2_O_2_ in water: The synergistic effect of adsorption. Sci. Total Environ..

[25] Li J., Zhang S., Chen C., Zhao G., Yang X., Li J., Wang X. (2012). Removal of Cu(II) and Fulvic Acid by Graphene Oxide Nanosheets Decorated with Fe_3_O_4_ Nanoparticles. Acs Appl. Mater. Interfaces.

[26] Fan L.L., Luo C.N., Sun M., Qiu H.M., Li X.J. (2013). Synthesis of magnetic beta-cyclodextrin-chitosan/graphene oxide as nanoadsorbent and its application in dye adsorption and removal. Colloids Surf. B-Biointerfaces.

[27] Liakos E.V., Gkika D.A., Mitropoulos A.C., Matis K.A., Kyzas G.Z. (2020). On the combination of modern sorbents with cost analysis: A review. J. Mol. Struct..

[28] Sillanpää M., Ncibi M.C., Matilainen A. (2018). Advanced oxidation processes for the removal of natural organic matter from drinking water sources: A comprehensive review. J. Environ. Manag..

[29] Chen W.H., Wong Y.T., Huang T.H., Lin J.G. (2019). Removals of pharmaceuticals in municipal wastewater using a staged anaerobic fluidized membrane bioreactor. Int. Biodeterior. Biodegrad..

[30] Chen J., Gu B.H., LeBoeuf E.J., Pan H.J., Dai S. (2002). Spectroscopic characterization of the structural and functional properties of natural organic matter fractions. Chemosphere.

[31] Lin C.H., Chen W.H. (2021). Influence of water, H_2_O_2_, H_2_SO_4_, and NaOH filtration on the surface characteristics of a graphene oxide-iron (GO-Fe) membrane. Sep. Purif. Technol..

[32] Li Z.X., Shakiba S., Deng N., Chen J.W., Louie S.M., Hu Y.D. (2020). Natural Organic Matter (NOM) Imparts Molecular-Weight-Dependent Steric Stabilization or Electrostatic Destabilization to Ferrihydrite Nanoparticles. Environ. Sci. Technol..

[33] Marais S.S., Ncube E.J., Msagati T.A.M., Mamba B.B., Nkambule T.T.I. (2019). Assessment of trihalomethane (THM) precursors using specific ultraviolet absorbance (SUVA) and molecular size distribution (MSD). J. Water Process Eng..

[34] Farivar F., Yap P.L., Hassan K., Tung T.T., Tran D.N.H., Pollard A.J., Losic D. (2021). Unlocking thermogravimetric analysis (TGA) in the fight against “Fake graphene” materials. Carbon.

[35] Kar K. (2022). Handbook of Fly Ash.

[36] Konkena B., Vasudevan S. (2012). Understanding Aqueous Dispersibility of Graphene Oxide and Reduced Graphene Oxide through pK(a) Measurements. J. Phys. Chem. Lett..

[37] Zhao T., Chen R.Q., Wang J.P. (2020). A Mild Method for Preparation of Highly Selective Magnetic Biochar Microspheres. Int. J. Mol. Sci..

[38] Alizadeh A., Khodaei M.M., Beygzadeh M., Kordestani D., Feyzi M. (2012). Biguanide-Functionalized Fe_3_O_4_/SiO_2_ Magnetic Nanoparticles: An Efficient Heterogeneous Organosuperbase Catalyst for Various Organic Transformations in Aqueous Media. Bull. Korean Chem. Soc..

[39] Zhang J., Chen D.-D., Li L., Li W.-W., Mu Y., Yu H.-Q. (2016). Role of NOM molecular size on iodo-trihalomethane formation during chlorination and chloramination. Water Res..

[40] Yang Z., Yan H., Yang H., Li H., Li A., Cheng R. (2013). Flocculation performance and mechanism of graphene oxide for removal of various contaminants from water. Water Res..

[41] Erickson H.P. (2009). Size and Shape of Protein Molecules at the Nanometer Level Determined by Sedimentation, Gel Filtration, and Electron Microscopy. Biol. Proced. Online.

[42] Li Q., Snoeyink V.L., Mariñas B.J., Campos C. (2003). Pore blockage effect of NOM on atrazine adsorption kinetics of PAC: The roles of PAC pore size distribution and NOM molecular weight. Water Res..

[43] Zimmerman A.R., Goyne K.W., Chorover J., Komarneni S., Brantley S.L. (2004). Mineral mesopore effects on nitrogenous organic matter adsorption. Org. Geochem..

[44] Igwegbe C.A., Aniagor C.O., Oba S.N., Yap P.-S., Iwuchukwu F.U., Liu T., de Souza E.C., Ighalo J.O. (2021). Environmental protection by the adsorptive elimination of acetaminophen from water: A comprehensive review. J. Ind. Eng. Chem..

[45] Desai D., Wong B., Huang Y., Ye Q., Tang D., Guo H., Huang M., Timmins P. (2014). Surfactant-mediated dissolution of metformin hydrochloride tablets: Wetting effects versus ion pairs diffusivity. J. Pharm. Sci..

[46] Kumari K.S., Bandhakavi S. (2020). Development and validation of stability-indicating RP-HPLC method for the simultaneous determination of ertugliflozin pidolate and metformin hydrochloride in bulk and tablets. Future J. Pharm. Sci..

[47] Settimo L., Bellman K., Knegtel R.M.A. (2014). Comparison of the Accuracy of Experimental and Predicted pKa Values of Basic and Acidic Compounds. Pharm. Res..

[48] Chang E.D., Town R.M., Owen S.F., Hogstrand C., Bury N.R. (2021). Effect of Water PH on the Uptake of Acidic (Ibuprofen) and Basic (Propranolol) Drugs in a Fish Gill Cell Culture Model. Environ. Sci. Technol..

[49] Kong F.X., Liu Q., Dong L.Q., Zhang T., Wei Y.B., Chen J.F., Wang Y., Guo C.M. (2020). Rejection of pharmaceuticals by graphene oxide membranes: Role of crosslinker and rejection mechanism. J. Membr. Sci..

[50] Nam S.-W., Jung C., Li H., Yu M., Flora J.R., Boateng L.K., Her N., Zoh K.-D., Yoon Y. (2015). Adsorption characteristics of diclofenac and sulfamethoxazole to graphene oxide in aqueous solution. Chemosphere.

[51] Kimbi Yaah V.B., Zbair M., Botelho de Oliveira S., Ojala S. (2021). Hydrochar-derived adsorbent for the removal of diclofenac from aqueous solution. Nanotechnol. Environ. Eng..

[52] Vadivelan V., Kumar K.V. (2005). Equilibrium, kinetics, mechanism, and process design for the sorption of methylene blue onto rice husk. J. Colloid Interface Sci..

[53] Wu Y., Li Z.M., Chen J., Yu C.G., Huang X., Zhao C.Z., Duan L.F., Yang Y., Lu W. (2015). Graphene nanosheets decorated with tunable magnetic nanoparticles and their efficiency of wastewater treatment. Mater. Res. Bull..

[54] Yang T., Lin H., Zheng X., Loh K.P., Jia B. (2017). Tailoring pores in graphene-based materials: From generation to applications. J. Mater. Chem. A.

[55] Jiang L.-H., Liu Y.-G., Zeng G.-M., Xiao F.-Y., Hu X.-J., Hu X., Wang H., Li T.-T., Zhou L., Tan X.-F. (2016). Removal of 17β-estradiol by few-layered graphene oxide nanosheets from aqueous solutions: External influence and adsorption mechanism. Chem. Eng. J..

